# Barriers and facilitators to implementing simulation into pharmacy programs globally

**DOI:** 10.1186/s40545-023-00531-6

**Published:** 2023-02-21

**Authors:** Harjit K. Singh, Vivienne Mak, Keith Sewell, Daniel T. Malone

**Affiliations:** 1 Faculty of Pharmacy and Pharmaceutical Sciences, Monash University, 381 Royal Parade, Parkville, VIC 3052 Australia; 2 Keypath Education, Melbourne, VIC Australia

**Keywords:** Dispensing, MyDispense, Pharmacy education, Simulation

## Abstract

**Background:**

MyDispense is a simulation software developed by Monash University that has been utilized by over 200 institutions worldwide to educate pharmacy students. However, little is known about the processes by which it is used to teach dispensing skills to students and how they use it to facilitate critical thinking in an authentic environment. This study aimed to understand and investigate how simulations are used to teach dispensing skills in pharmacy programs globally, and to determine the opinions, attitudes and experiences of pharmacy educators towards MyDispense and other simulation software within their pharmacy program.

**Methods:**

Purposive sampling was used to identify pharmacy institutions for the study. A total of 57 educators were contacted, 18 responded to the study invitation, 12 were MyDispense users and 6 were non-users. Two investigators conducted an inductive thematic analysis to generate key themes and subthemes to provide insight into the opinions, attitudes and experiences towards MyDispense and other simulation software used specifically for dispensing within pharmacy programs.

**Results:**

26 pharmacy educators were interviewed, of which 14 were individual interviews and four were group interviews. Intercoder reliability was investigated and a Kappa coefficient of 0.72 indicated substantial agreement between both coders. Five main themes were identified: “dispensing and counseling”, which encompassed discussions about how dispensing techniques were taught, the time allocated for students to practice their skills and the use of software other than MyDispense; “description of MyDispense use” includes discussions about the setup of the software, how dispensing skills were taught prior to using MyDispense as well as its use in student assessments; “barriers to MyDispense use”, covers discussions about the obstacles users have faced; “facilitators to use MyDispense”, includes discussion about the various motivators to using MyDispense and lastly “future use and suggested improvements” of MyDispense are covered by the interviewees.

**Conclusion:**

The initial outcomes of this project evaluated the awareness and utilization of MyDispense and other dispensing simulations by pharmacy programs globally. By addressing the barriers of use, promotion of the sharing of MyDispense cases can assist in creating more authentic assessments, as well as improving staff workload management. The outcomes of this research will also facilitate the development of a framework for MyDispense implementation, thus streamlining and improving the uptake of MyDispense by pharmacy institutions globally.

**Supplementary Information:**

The online version contains supplementary material available at 10.1186/s40545-023-00531-6.

## Background

High-fidelity simulation can incorporate psychological and engineering technology to provide students with realistic learning and/or assessment experiences in a controlled environment [1]. Implementation of high-fidelity simulation to teach and assess students provides an evolving understanding of learning and competency in a simulated environment resembling their future professional practice [2]. Being a formative teaching and assessment strategy, it also enables consideration of student perceptions and experiences [2]. A well-known advantage of incorporating simulation software into pharmacy education is that students can make mistakes without real-life consequences [3]. In addition, the qualities of simulation software enable students to re-attempt cases infinitely, empowering them to gain firsthand experience of the outcomes of their decision-making in a safe environment, facilitating their appraisal of choices. Therefore, simulation can be used to teach a number of pharmacist-relevant topics and skills, including pharmacotherapeutics, patient care processes, patient-centered care, and interprofessional team-based care [4].

The 2019 Coronavirus Disease (COVID-19) pandemic had a significant impact on pharmacy education globally. Strained health care systems limited the operation of experiential learning placements, while the forced self-isolation of people and closed borders between counties and states meant that at times both staff and students may have been displaced or isolated [5]. Consequently, there was a desperate need for Universities and educators to adapt to and develop not only innovative communication methods, but also teaching methods to ensure pharmacy students could continue to feel engaged, learn and practice their skills in a way which emulates real practice. One such pedagogical technique is the use of high-fidelity simulation software [6]. MyDispense is a free online pharmacy virtual high-fidelity simulation software that was developed for pharmacy education [7] by the Faculty of Pharmacy and Pharmaceutical Sciences at Monash University Australia [8]. MyDispense was formally introduced into the Monash pharmacy curriculum in 2011 [8] and has since been updated multiple times to enhance user experience and application [9]. The software allows users to customize the assets used in MyDispense, including patient names and addresses, prescribers, medications, prescriptions and dispensing labels in order to comply with the pharmaceutical regulations and cultural norms of their region. Pharmaceutical references accessed by students via MyDispense can also be tailored to each institution.

Since its inception, MyDispense has been deployed to over 200 institutions globally [9]. The software enables pharmacy students to practice dispensing, using various community and hospital pharmacy case-based scenarios of varying complexity, from beginner to highly advanced, in a safe and a realistic environment without the potentially life-threatening consequences of a real-life error [7]. These simulations enable students to gain experience in pharmacist–patient interactions such as filling prescriptions, helping patients with self-care needs, validating the work of virtual colleagues to ensure that medications are accurate, legal, and safe before dispensing, as well as retrieval of medications from shelves/fridges/safes, provision of medicines, and providing appropriate counseling [8]. As such, its innovative design means that it can be adapted and successfully used to teach dispensing theory and practices, as well as provide immediate feedback upon exercise completion [8].

Although MyDispense is used by many pharmacy institutions globally, little is known about how pharmacy educators use it to teach dispensing skills to their students and how MyDispense is used to facilitate skills practice and development. Furthermore, there is a gap in the literature about the process of how MyDispense has been implemented in Pharmacy institutions, or what attitudes and perceptions educators have regarding the use of MyDispense. The purpose of this research was to extend the understanding of, and investigate how simulations such as MyDispense are used to teach dispensing skills in pharmacy programs globally. The aim was to determine the opinions, attitudes and experiences of pharmacy educators regarding MyDispense and other dispensing simulation software used within pharmacy programs.

## Methods

An exploratory technique was adopted to interview representatives from pharmacy programs globally. Ethics approval for this study was received from the Monash University ethics committee (2022-31548-73284).

A purposive and snowball sampling strategy [10] was implemented in order to devise a list of pharmacy institutions from around the world that are high, intermediate and low users and non-users of MyDispense. High users of MyDispense were defined as those that utilized the software for greater than 1000 activities, intermediate MyDispense users utilized the software for 100 to 1000 activities and low users utilized the software for less than 100 activities. Contact and activity details of representatives from the pharmacy institutions shortlisted were identified from the Monash University MyDispense database and from online public domain sources. In all 36 MyDispense users and 21 non-users that were not using MyDispense were approached via email to participate in the study. MyDispense users invited were from Australia (AUS), New Zealand (NZ), the United States (US), the United Kingdom (UK), South Africa, Philippines, Libya, Saudi Arabia, Hong Kong (HK), Malaysia, Indonesia and Zimbabwe, while non-users were identified from AUS, NZ, US, UK, Vietnam and Malaysia. Non-users were included in the study as they would provide us with valuable insight about other pharmacy simulation programs being used globally, how they are used to teach dispensing skills to students and how they differ from MyDispense. This would enable us to capture areas in which MyDispense could be improved to enhance user experience and encourage utilization.

A study investigating the use of MyDispense generally across only the United States (US) has previously been conducted [11]. The interview guide was based on this US study, but the current study is novel as it investigated the use of MyDispense and other pharmacy simulation programs utilized by pharmacy institutions globally.

Questions in the guide consisted of six main questions and follow-on prompts and included characteristics about where in the pharmacy degree dispensing is taught, how dispensing is taught, how dispensing skills are assessed, and the utilization of dispensing simulation software (if any). The interview guide for the study was piloted internally on two academic staff; one who was well versed with MyDispense and one that was not familiar with MyDispense. Interviewees were probed through the use of follow-up questions to encourage elaboration and clarification of responses that were otherwise unclear or vague, ensuring that the data gathered would be appropriately interpreted and analyzed. Subsequent to the pilot, minor changes were made to the interview guide to ensure that details about how dispensing is taught could be accurately captured. The results from the pilot were not included in the final analysis.

Semi-structured interviews were conducted from April 2022 through to June 2022 until data saturation was reached, whereby no new data revealed any additional insights or information related to the topics of interest. The participant consent form provided an overview of the study aims and the types of questions that would be asked. Interviews were undertaken by a member of the research team via Zoom at a time convenient for the participants.

Although the initial study design proposed one-on-one interviews with participants, some MyDispense non-user institutions stated that they had implemented the software across their pharmacy degree and requested to include colleagues in the interview panel, hence group interviews were conducted in these cases.

Digitally recorded interviews were transcribed verbatim and checked for accuracy by one team member. Data management was facilitated by NVivo (Release 1.3) (QSR international 2020). A thematic analysis method was chosen to analyze the data [12]. Two investigators read the first four interview transcripts and independently analyzed the data, inductively coding the data to generate a list of codes [13]. Differences in coding were reviewed for each interview, and discrepancies resolved code-by-code and a new coding paradigm was developed. In the second round, both investigators reviewed and re-coded the first four interviews according to the new coding paradigm while looking for new codes. The individual coding files were then merged to generate a final coding scheme and the codes were categorized to generate key themes. Inductive thematic saturation was reached on the basis of no new codes being observed in the data [14]. After each phase, intercoder reliability was calculated using Cohen’s kappa. A Cohen’s kappa of 0.70 was set as a minimum value to achieve, considering values of > 0.70 are typically used in exploratory research [15] and indicate substantial agreement [16]. To achieve this, the two investigators met four times to discuss coding to improve intercoder reliability. The coding scheme was reduced by excluding codes that were not coded often or at all, and codes similar to one another were also combined. To reduce ambiguity, the investigators also decided to consistently code text segments of up to three lines and to include whole sentences. In the third round, the two investigators re-coded the first four interviews using the updated coding scheme and rules. However, a kappa score below the target indicated the investigators meet again to discuss the coding scheme and rules. In the fourth round, the investigators combined additional codes and coded one interview together before coding four interviews independently. When intercoder reliability was > 0.70, the remaining interviews were coded by one investigator according to the final coding scheme.

## Results

In total, of the 57 educators who were contacted about the study, 18 responded to the invitation. Of these, 12 were MyDispense users (seven were high users of MyDispense, one was an intermediate user of MyDispense and four were low MyDispense users) and 6 were non-users. Subsequently, 26 pharmacy educators were interviewed, of which 14 were individual interviews and four were group interviews (ranging from two to four participants; Table 1). Interviewees were from various pharmacy institutions worldwide; eight interviewees were from Australia, five were from New Zealand and 13 were from other countries (Table 1). A diverse range of educational roles was also evident.

**Table 1:** Characteristics of pharmacy educators interviewed for the study

Interview no.	Country	MyDispense user	User type	Requested MyDispense account post-interview
01	Australia (Queensland)	Yes	Low	N/A
02	Australia (Queensland)	Yes	Low	N/A
03	Brunei	Yes	Low	N/A
04	Australia (Sydney)	Yes	High	N/A
05	South Africa	Yes	High	N/A
06	Libya	Yes	Intermediate	N/A
07	Australia (Victoria)	No	N/A	Yes
08	USA (West Virginia)	Yes	High	N/A
09	New Zealand (Auckland)	Yes	High	N/A
10	New Zealand (Otago)	No	N/A	Yes
11	United Kingdom	Yes	High	N/A
12	Germany	Yes	Low	N/A
13	Australia (Victoria)	Yes	High	N/A
14	Australia (NT)	No	N/A	Yes
15	Australia (ACT)	No	N/A	N/A
16	United Kingdom	No	N/A	Yes
17	Philippines	Yes	High	N/A
18	Australia (Victoria)	No	N/A	Yes

After the final round of coding, Cohen’s kappa for intercoder reliability was 0.72, indicating substantial agreement between coders [16]. Analysis of the interview transcripts revealed the emergence of five main themes: “dispensing and counseling”; “description of MyDispense use”; “barriers to MyDispense use”; “facilitators to use MyDispense” and “future use and suggested improvements” (Table 2). Subcategories are italicized in the following text and a quotation representative of each subcategory is also provided. Each interview was given a number, which is given after each quotation. Exemplar quotes for each subcategory are listed in Additional file 1: Appendix 1, Additional file 2: Appendix 2, Additional file 3: Appendix 3.

**Table 2:** Themes and subthemes

Main theme	Subcategory (*n* participants)
Dispensing and counseling	Dispensing techniques (18)
	Time allocated to practice (13)
	Other dispensing software (11)
Description of MyDispense use	Initial setup (8)
	Previously taught dispensing (6)
	Application (12)
	Frequency of use (6)
	Dispensing assessments (10)
Barriers to MyDispense use	University costs (8)
	Academic staff time (6)
	Lack of training (4)
	General difficulty in use (8)
	Lack of collaboration (7)
	Lack of realism (3)
	Difference in jurisdiction and drug nomenclature (4)
	Sustainability (4)
Facilitators to use MyDispense	Cost (7)
	Training (9)
	Promotes collaboration (8)
	Realistic environment (10)
	Controlled drug function (2)
	COVID (9)
	Cultural representation (5)
	Easy to use (7)
	Engaging experience (3)
	Saves staff time (6)
	Student feedback (8)
Future	Plans (9)
	Suggested improvements (9)

### Dispensing and counseling

The first main theme that arose from the interviews with MyDispense users and non-users was dispensing and counseling. This theme encompassed discussions about *dispensing techniques, time allocated to practice* of dispensing and counseling as well as the utilization of *other dispensing software* not including MyDispense.

Each interview included some insight into how dispensing skills were taught within respective pharmacy programs. More specifically, this related to *dispensing techniques* such as handling prescriptions, conducting clinical and legal checks, resolving clinical issues, data entering, label preparation and counseling. Some participants who were MyDispense users indicated that they solely utilized MyDispense for teaching dispensing techniques. Some of these users appreciated the barcode scanning feature within MyDispense that is used to verify correct medication selection and minimize the risk of labeling the incorrect product. Other users indicated that they used MyDispense in conjunction with other software to support teaching techniques such as group work and face-to-face interactions. There were differences in when dispensing skills were first introduced and taught in each pharmacy program, but all participants agreed that students must be competent in dispensing skills by the end of their final year.

“… I guess there’s the more mundane side of the dispensing where it’s product selection and label creation checking cal’s, doing the scan, which is now requirement, or at least legally pharmacies are required to have this scanning technology and MyDispense has that built in” [01].

With regard to the *time allocated to practice* dispensing, this varied considerably among the pharmacy programs depending on whether a dispensing simulation was utilized and its accessibility. It is important to note that for MyDispense user institutions, students can have access to the software remotely as long as they have internet access, which allows practice of additional cases and techniques in their own time. Some MyDispense non-user institutions could provide students access to their chosen simulation software onsite outside of allocated class time. The importance of practicing dispensing prior to commencing pharmacy placements or student pharmacist roles was a commonality across interviews.

“Like the second year, when I suppose for a second year they’re so new and we assume that they don’t have jobs and pharmacy yet maybe some of them do, but a lot of them wouldn’t have at that point, and so, and they have 3 three hour dispensing labs and three two primary healthcare workshops and so.” [09].

It was evident from the interviews that those who do not utilize MyDispense generally used *other dispensing software* or simulation platforms. This included software typically utilized within community pharmacy practice such as Fred Dispense [17] in Australia and Z Software [18] in New Zealand. Other Pharmacy institutions who did not use MyDispense opted to use other computer simulation software for Pharmacy education purposes. Examples include SimPharm [19] and Script-Ware [20], which, similar to MyDispense, are also web-based simulation platforms that expose students to Pharmacy scenarios that mimic reality [21]. Another software that was utilized by MyDispense non-users was an educational electronic health record called EHRGo [22], available to educational institutions for teaching and learning purposes [22]. This software varies from Pharmacy simulation software as it is designed to be used by students from a number of disciplines. It exposes students to simulated patient cases, health information management, dietetics, interprofessional education content as well as a medication dispensing system, specifically built for Pharmacy education [22].

Typically, these software programs were associated with an initial license cost, and may involve ongoing maintenance costs. Access to updated versions also required additional expenditure, though most educators were not aware of this. Reasons for using other dispensing software included preparing students for future practice, or it had already been implemented and used by the institution before the educator commenced. The industry-used software were recognized to have limitations such as the inability to create mock patient profiles for teaching purposes or simulate real life as they lacked the element of patient communication. Some also had limited capacity to be available remotely to students.

“Students can always practice dispensing from home if they wish to, apart from the on-campus sessions, with the simulator provided by the government called FRED dispense. They can also do so through other software like EHRGo. You can dispense on EHRGo if you want to…” [07].

### Description of MyDispense use

The second main theme that arose from the interviews was description of MyDispense use. This theme included a description of the *initial setup* of MyDispense by users, accounts of how students were *previously taught dispensing* prior to using MyDispense, the *application* of MyDispense in various topics, units or courses throughout the Pharmacy degree*,* the *frequency of MyDispense use* as well as the use of MyDispense for* dispensing assessments.*

Of the 12 interviews with people from MyDispense user institutions, only eight described the *initial setup* of the software. These educators were either directly involved in the process or were currently accountable for updating it. The setup of MyDispense typically involved liaison with the information technology (IT) team at the institution and the MyDispense team at Monash University. To facilitate ample storage and security, some educators described the need for an additional server or cloud space which was organized by the IT team. Overall the setup of MyDispense was described as seamless by the educators.

“No. it’s all approved within the school, but we are part of a greater Faculty of Medicine and health, so when it came to switching over to the new server I had to go up to a faculty level to get the support that I needed to do that kind of thing”. [04].

When describing the application of MyDispense, many users also discussed how they *previously taught dispensing*. This included the incorporation of industry dispensing software, a scanner, and a label printer as part of dispensing laboratories. In some pharmacy institutions, students also had access to a mock pharmacy including shelves of medicines. During dispensing laboratories students would dispense mock prescriptions. However, this process was limited as students could only complete these classes face-to-face and were confined to dispensing an allocated number of prescriptions. The implementation of MyDispense was described as improving teaching and learning as students could practice their dispensing skills using MyDispense an infinite number of times. In addition, dispensing laboratories were not limited to face-to-face classes as MyDispense was available to students remotely.

“So, we never had a kind of simulation software before and before we had dispensing software like a pharmacy might use in actual practice…and so it was useful for kind of the process of making labels and but then everything else was extremely resource intensive so buying all the medicines there’s obviously a cost associated there and then they have a limited experience so we were finding more and more of our medicines were becoming expired.” [11]

Although MyDispense is predominantly used to teach dispensing theory and skills, many educators outlined the *application* of MyDispense within a number of different topics. This included various primary care conditions such as cardiology, hypertension, arrhythmias, coronary heart disease, smoking cessation, pain management, hospital pharmacy, herbal medicines, halal medicines, calculations and experiential placements.

“We cover dispensing, also prescription checking, calculations, responding to symptoms, yeah herbal medicines, even halal medicines are covered. We have some interesting use cases on our halal medicines and also on the medicine management during Ramadan yeah.” [03].

With regard to the *frequency of MyDispense use* within the courses specific to dispensing, this varied among institutions. In addition, dispensing skills were not solely taught using MyDispense, but rather were also covered in lectures.

“MyDispense is started from day one of each rotation until the end of the rotation.” [06].

MyDispense also has assessment capabilities, and 10 of the 12 MyDispense users interviewed for the study used it for the purpose of *dispensing assessments.* This depended on the institution's ability to manage the online assessments as well as the educator’s capacity to build and test assessments within the software prior to examination. MyDispense non-users all used other simulation software to assess students' dispensing skills. Both MyDispense users and non-users engaged a form of virtual simulation in conjunction with a face-to-face simulation method such as Objective Structured Clinical Examinations (OSCEs) to test students.

“… with MyDispense there’s like that simulated patient that incorporates that communication skills. So, before this our OSCE was that communication skills separate to dispensing skill set…so I think it’s beneficial to have this it’s more added value to have this MyDispense software implemented in our teaching and learning module because of the additional skills that the student can be taught and practice, actually, even if you dispense it wrong is still don’t kill the patient right.” [03].

### Barriers to MyDispense use

Another main theme that arose from the interviews was barriers to MyDispense use. This included discussions about *University costs* associated with the implementation of MyDispense*,* as well as *academic staff time* commitment involved*, lack of training* available for users, *general difficulty in use* of MyDispense, the *lack of collaboration* users experience, *lack of realism* associated with the software, problems with *drug nomenclature*, problems with sharing MyDispense case templates with regions bound by *different jurisdictions,* and the continued *sustainability* of the software as cost free.

Although MyDispense is a free simulation, many users expressed that there were additional *University costs* associated with its implementation. These costs were related to the time required by professional staff such as those in IT for their involvement with the setup and ongoing support to use MyDispense. In addition, a few institutions also hired staff to assist with the administrative tasks associated with MyDispense such as enrollment of students into the system, creating a drug library; which involved taking pictures of medications, and the development of MyDispense cases. Other costs incurred by some institutions included those associated with upgrading internet bandwidth to support MyDispense and the use of proctoring systems to support equitable assessment practices while utilizing MyDispense.

“When we started off, I think our internet was a bit overwhelmed as for the server I guess was overwhelmed with the number of students that were taking are using MyDispense at the same time…So, in terms of upgrades I think we did have to shell out some of technicalities in terms of increasing bandwidth upgrading internet and facilities to cover all of the students using the internet, at the same time” [17].

The most expensive cost was *academic staff time* regarding learning how to use MyDispense, training colleagues, creating a drug library as well as building and testing cases that had been created within the software.

“Not from a monetary point but I suppose one of the things that really needs a champion and we have had a champion in our department of actually doing the graft around us and, so you need people, and the people need time and this is probably the most stretched resource really.” [09].

Interviewees also mentioned *lack of training* pertaining to building and updating cases within the software as another barrier associated with MyDispense use. Problems described were mainly associated with the prescription setup within MyDispense, including the upload of doctors’ signatures, which needed to be the correct size and resolution in order to be accepted by the MyDispense system. The setup of multiple ingredient medications and ancillary labels were also considered challenging tasks but imperative to developing cases which reflect real pharmacy practice.

“…I think the second thing would be expanding the portfolio of medications that we can make script prescriptions from…for example, sometimes we’re looking for a generic alternative to demonstrate that difference in from the known brand to perhaps a generic and there’s not always a generic in the system and then to actually set that up…we’ve had to seek help because of the images that are required and the scanning and those sorts of things for the higher levels of the course which is more sophisticated…I have found that cumbersome if it’s not already in the system.” [04].

There was also some *general difficulty in use* associated with MyDispense, this concerned the enrollment of students into the system as well as the authorization of MyDispense to conduct assessments. One MyDispense user also discussed a lack of computer literacy among their students, which limited their capability to navigate the software. Similarly, another interviewee mentioned that staff would often walk students through MyDispense over the phone to facilitate progress through cases when they would get stuck with the system. There were some interviewees that described students perceiving MyDispense as a game, which potentially limited its utilization in the application of knowledge.

“So that we did have in 2020 some feedback that they thought it was a game. We gave them videos and PDFs of how to access and how to navigate MyDispense, but because they saw the avatars and things they thought it was a game, they didn’t really take it seriously and they didn’t understand how to optimize it and they didn’t even get to the feedback PDF at the end.” [09].

A key consideration of disseminating MyDispense as a free software is the sharing of cases among user institutions, thus showing altruism and easing staff workloads. However, many users described being unaware of this, influencing a *lack of collaboration.* Some interviewees stated that they would not appreciate sharing MyDispense content with other pharmacy institutions within their region as they did not partake in the initial setup.

“I suppose it would be a resource thing, I would have to talk to the powers above to see if they will be willing for us to share. Because certainly building up that product bank and all the images like I guess it’s been a barrier to using it more, and so, if there was some way that you could split that sort of stuff with another provider there would be some benefits to that….” [09].

MyDispense cases are designed to mimic real pharmacy practice scenarios. However, some users described there was a *lack of realism* within the software. This was typically related to comparisons made between the software and typical pharmacy practice which is ultimately a face-to-face interaction and cannot be substituted. For this reason, one institution described that they will be working towards more face-to-face interaction and limiting the utilization of MyDispense. Comments were also made about a superior competing simulation software used in pharmacy education as it involves collaboration and interaction with other health professionals, however currently interaction with virtual patients within MyDispense is limited to selection of questions to ask a patient, thus is not the same as artificial intelligence.

“MyDispense has definitely helped. I think there are benefits to keeping it as a supplementary activity. I think for our assessment, I think we need to move back to simulating. You know the actual practice of dispensing skills and counseling and face to face as soon as possible.” [01].

During interviews, the management of MyDispense drug libraries to reflect harmonized *drug nomenclature* was described by some users as a challenging task. In addition, sharing of MyDispense case templates with regions bound by *different jurisdictions* limited usability as changes needed to be made to ensure they reflected policies and regulations specific to the area.

“When I took over everything was Melbourne addresses or Victorian doctors. Which from a jurisdiction and legal point of view, has some implications, especially when we start talking about controlled substances…” [04].

Another barrier to using MyDispense was the perceived lack of *sustainability* of the product. Although MyDispense is a free software, and despite it being publicly stated on the MyDispense website [9] that it will always be shared with no fee, many users wondered how long it would be free, describing that their colleagues also felt similarly and hence denied learning more about it. MyDispense non-users also raised concerns about this.

“And yeah, I mean everybody at the university when we’ve spoken about it and I’ve been teaching and learning and forums and they you know they will say, and this is free, and you know they're all very … concerned about the sustainability, how long this is going to remain free.” [05].

### Facilitators to implementing MyDispense

Facilitators to implementing MyDispense arose as another main theme. This encompassed conversations about limited *costs* associated with implementing the software, *training* involved*, collaboration* with other MyDispense user institutions*,* how MyDispense portrays a *realistic environment* to students*,* benefits of the *controlled drug function* within the software*,* the role of the *Coronavirus pandemic 2019 (COVID-19)* in supporting MyDispense implementation*, cultural representation* of ethnic groups in MyDispense, that the software is *easy to use* for both staff and students, and provides an *engaging experience* for students and also *saved staff time.*

A key facilitator to implementing MyDispense was that the simulation was free to use. Some interviewees stated that this saved their institution money as they would not have to incur the *cost* for alternative simulation software, yet still reap many of the benefits.

“So, we don’t use the old dispensing software anymore, and it was quite expensive as well. That was another plus side of MyDispense obviously being free software is that it freed up resources for us to use elsewhere …” [11].

Many of the MyDispense users described having had a positive experience in the *training* that they received from the MyDispense team as well as fellow colleagues who used the software. In addition, these connections also facilitated the development of country specific customized versions of MyDispense thereby enhancing teaching, student usability and understanding of pharmacy knowledge. To further enhance the student user experience, several institutions outlined the development of in house MyDispense training for students.

“So every batch… one day for them to teach them like, as you said, a lecture on how to deal with MyDispense. Starting from how to enter into the database and how to give them the password, the username and so on. And we practice and train them, step by step, this is the computer screen, how to search the patient’s name, and history, and this is how to check for the prescription, this is how to check for drug interactions. This is how to go through the questions for the patient and the questions for the doctors as well…” [06].

Conference attendance was the principal means by which interviewees initially became aware of MyDispense and its capabilities. During this time connections were made with the MyDispense team to initialize the software. Participants from some institutions described liaising with colleagues at partner institutions for the purposes of MyDispense training which also *promotes collaboration* on development of cases and research projects.

“We could extend that collaboration to research. This could open up many opportunities for us as well, so from just one software it could. It could link us to other countries and also other opportunities, I think so that’s a way forward” [03].

During the interviews MyDispense users expressed that a key advantage of its application within Pharmacy education is its ability to provide a *realistic environment* thereby preparing students about what to expect on experiential placements and can also be used as a replacement. Users described that the software exposes students to and facilitates the development of a number of pharmacist-relevant skills such as reading prescriptions, and learning medication names, the familiarization with the appearance of medications and their packaging, ancillary labels (and where to affix them), and dispensing labels. Furthermore, unlike industry software, MyDispense incorporates patient interactions and pharmacy scenarios alongside the dispensing process. This includes asking patients questions, communication with healthcare professionals and practicing counseling skills. Users were also enthused by the feedback feature within MyDispense.

“I think it gives them a feel for the real-world pharmacy a little bit in terms of you know kind of what to expect situations that might arise as they work in a pharmacy. You know, as with any simulation not all aspects are going to be able to be simulated but, for the most part, I think it does a pretty good job…” [08].

MyDispense also includes a controlled register for entry of medicines into and out of the dispensary and also simulates where and how these medications are stored and accessed. Interviewees described another facilitator to the utilization of MyDispense, the *controlled drug function* can be used to assist the development of student knowledge and skills about regulations and processes involved in dispensing a controlled drug.

“… my favorite part is the drug part, so I like how they will separate them, you know controls and non-controlled. Because that’s very much so, how it is in the pharmacy and so there is like a safe, where they keep you know your controlled substances and the students will have to know ‘Oh, this is a controlled substance’, I need to get it from the safe.” [08].

A significant facilitator that expedited the use of MyDispense by many institutions was *COVID-19*. Many MyDispense users reported that the lack of face-to-face classes motivated them to identify alternative ways of teaching and promoting student engagement. Interviewees described how MyDispense fitted this profile as well as assisted during the COVID-19 pandemic by simulating student placements that could not be offered because of the pandemic.

“At least we have found a solution during the pandemic area…at least, we found the solution, other universities are still struggling at that point.” [06].

An added benefit of MyDispense that was discussed during the interviews was its customizability, and the inclusion of avatars representing various cultural and ethnic groups. This was considered a valuable attribute by educators as it made MyDispense engaging for students. In addition, it made education about topics such as halal medicines and Ramadan more realistic by exposing students to various cultural experiences that they may not have otherwise been exposed to. The ability to customize MyDispense means that it can be modified to include country specific details such as prescription types, medicines and policies, thus adding an element of *cultural representation.*

“We introduced the halal medicine cases only this semester. We wanted to expose different cultural experiences to the Philippines students, so I think the students felt like it was very relevant… It’s nice that they incorporated people with scarf I mean on my perspective, so it's good like oh, I want to talk about a Muslim, so I want an appropriate picture to incorporate so when trying to find the right icon or Avatar …you provide it is more inclusive I find so that's kind of nice and like being I’m a Muslim myself, so if I want to add some cultural counselling in a way for our setting so that’s what I like about MyDispense well.” [03].

Supported by the availability of online manuals and videos for users, MyDispense was described as being quite user friendly and *easy to use* for both staff and students. In addition, users made specific mention that no issues with compatibility of the software to devices and browsers were apparent. Educators also stated that they had not received any complaints from students about the software and that it was always functional.

“I think I looked at your online video as well, there were some helpful online videos I think I went to and I watched a few of those to you know pick up my skills, but no, no formal training, it’s quite user friendly.” [01].

MyDispense users described that the incorporation of software within teaching activities made it a more *engaging experience* for students. Interviewees also noted the participation of students in MyDispense activities was also good, and could be tracked within the software. The introduction of preparatory MyDispense activities prior to face-to-face classes encouraged students to come prepared, improving their interactions.

“I guess anecdotally I think they’ve liked it… I think, to me, the actual engagement is the key like you know I’m actually seeing the students both externally and internally logging in and tapping through those activities, and I think that’s probably the key driver that I’m getting you know a good level of participation.” [01].

The MyDispense software includes a prompt feedback feature that can be utilized to provide students with comments and answers upon completion of a case. This was described as being beneficial as it *saved staff time* from having to provide students individual feedback. Although setting up cases and feedback in MyDispense took considerable time, once it was set up, it only occasionally required updating, which also freed up staff to do other academic tasks.

“… once we’ve got the scenarios set up and running, there are things that you can do that free us to do other things in terms of…On one level it obviously takes staff input in the beginning, but once it's up and running, it frees staff actually to do other things yeah.” [05].

During the interview, educators were asked about their *student feedback* pertaining to MyDispense, the majority stated that the students enjoyed their experience and felt what they were doing within MyDispense was relevant to pharmacy practice.

“The student feedback certainly seems like they enjoy it.” [04].

### Future use of MyDispense

The last main theme that arose during the interviews with MyDispense users and non-users was about future use. This included discussions about the interviewees’ *plans* pertaining to MyDispense as well as *suggested improvements* of the software.

When questioned about their future *plans* on the use of MyDispense, most users stated that they would continue to use the software as part of their teaching, some also hoped to utilize the assessment features within the software. On the contrary, one user stated that although they would continue to use it as supplementary material, they would be more inclined to revert to face-to-face interaction. A few users also indicated they would be collaborating with other institutions that use MyDispense for the purposes of research projects. Some users also suggested plans to liaise with the MyDispense team at Monash to customize the software to include behind the counter ‘pharmacist only medicines’ and point of care testing. There was also mention of introducing the software to registered practitioners as existing industry software within their region was not as sophisticated as MyDispense. Of the six MyDispense non-users interviewed, five requested access to MyDispense for future teaching purposes.

“The next thing that we, we can do is that maybe we can introduce this also to the pharmacy practitioners, because I think this can really influence them later on in terms of our pharmacy practice in the Philippines, so it’s like having all those patients recording and also having all those that we didn’t actually practice here in the Philippines, so we can actually have up there, later on.” [17]

Although there was mostly positive feedback about the software, interviewees made some *suggested improvements* including the incorporation of voice interaction, the ability to modify rules for controlled drugs, and universal system updates of drug nomenclature and the development of a generic drug library, the incorporation of interprofessional education, and ongoing training or communication of MyDispense updates.

“I feel like we’re not updated often enough, so I think I feel like the product is developing, perhaps the depth of the activities are developing and I don’t know about it, so I think that if I had to change anything and it’s not about software it’s about more about the two way communication between the MyDispense team and the institutions using it. is, I think we need to have a little bit more of an understanding of the changes that have been made in scope and that sort of thing. That would be one thing I would improve. Yeah it doesn’t even have to be in person and a newsletter even bulletin ones per semester.” [04].

## Discussion

The aim of this project was to investigate how dispensing skills are taught in pharmacy institutions globally and dispensing programs like MyDispense are used. Five key themes emerged from this study: “dispensing and counseling”; “description of MyDispense use”; “barriers to MyDispense use”; “facilitators to use MyDispense” and “future use and suggested improvements”.

Dispensing is inherent in pharmacy programs [8], and as such there was considerable overlap in how dispensing and counseling was taught among MyDispense users and non-user institutions. Dispensing techniques (including handling prescriptions, conducting clinical and legal checks, resolving clinical issues, data entry, label preparation and counseling) were taught at various stages in Pharmacy programs to meet dispensing competencies. In each instance, dispensing theory is taught and practiced by students using a dispensing software that is available to students on campus. Web-based software, including MyDispense, can be accessed remotely and thus has the advantage of enabling students to practice their skills anywhere and at any time. Some MyDispense users indicated that this was particularly advantageous for students who did not routinely work in a community pharmacy to practice and refine their skills. The use of MyDispense in pharmacy education, particularly if made available so that students can practice at a time of their choosing, would be beneficial to learners, considering evidence to support students controlling their study time based on self-regulation, whereby learning is based on the student self-monitoring, self-instruction, and self-reinforcement [23]. MyDispense can also assist students to develop their counseling skills, which are inherent to the role of a pharmacist. Studies have reported that qualified pharmacists lack the ability to generalize counseling skills taught and practiced during their university education when they are not provided with sufficient opportunity to practice their skills in a context that mimics real life [24]. In addition, simulated patient interactions and prompt performance feedback improved pharmacist’s counseling skills [24]; which were practical features frequently implemented by MyDispense users. Moreover, MyDispense users acknowledged that these attributes improved student autonomy as students came prepared for classes which accommodated more time to focus on the development of face-to-face skills such as counseling.

The usability of technology in higher education is imperative to its integration [25]. The Participants in this study described the initial set up of MyDispense as straightforward. Though it was evident from interviews that communication with IT teams within the institution and the MyDispense team were crucial to this, as it would be for the setup of other pharmacy education software and industry software. Various pharmacy simulation software can be used to integrate dispensing theory with a number of primary health care topics and can also be combined with face-to-face simulations techniques such as workshop role-plays, teaching-OSCEs or OSCEs which is a valuable quality. However, only web-based simulation software such as MyDispense enabled students the opportunity to practice their pharmacy skills independently.

Building a community of practice has been an integral philosophy of MyDispense and was a key driver in the decision to keep the software free and encourage users to freely share their work [26]. Despite this, sharing of cases was one of the main barriers to MyDispense use described by the interviewees. This sharing feature was something that some interviewees were unaware of, while others were apprehensive to share and considered the materials they had created as classified. In addition, cases created by institutions may not be directly relevant to others because of differences in their definitions of formative and summative assessment strategies [27], regional jurisdiction, policies and cultural differences. However, it should be noted that the underlying framework of the case could simply be updated which alleviates much of the heavy lifting required to build cases from scratch. Furthermore, upon collaboration with the MyDispense team, the software can be customized to suit regional and institutional needs [7] thereby saving staff labor time and costs.

Other facilitators to implementing MyDispense identified in this study included the variety of avatars within the software. It was noted that the avatars could portray patients with a range of symptoms of diverse health conditions, cultural and ethnic backgrounds. In addition, many interviewees mentioned that students could interact with MyDispense avatars by asking questions and learn to provide culturally sensitive counseling as appropriate. It has been shown that exposure to simulated-patient activity is superior at enhancing student’s cultural competency than written case-scenarios or lectures alone [28]. This is another attribute of MyDispense that makes it superior to using a pharmacy industry software in the educational setting. Moreover, MyDispense also exceeds industry software as it includes a simulated dispensary and also provides prompt feedback to students upon completion of cases. Although it has been shown that there is no significant difference in the grades of students who received prompt feedback compared to those that receive delayed feedback [29], it would be reasonable to suggest that access to timely feedback provides students with explanatory and corrective feedback that may improve their learning [8].

High-fidelity simulation use within health professional education is expensive and can cost an average of US$30,000 [3]. This fee does not include the additional cost of maintenance, training, and technical support associated with the software [3]. However, unlike simulation programs used in the pharmacy industry and other pharmacy education software programs, there are no license costs or direct maintenance costs associated with MyDispense. Despite this, some users described occasional costs such as the purchase of cloud storage space, proctoring systems to facilitate authentic assessments, and the time and equivalent cost associated with the development of cases and drug libraries. It is important to note that other than the latter, all expenses are optional as they do not limit the usability of the software.

There were reports from participants that the lack of voice integration affected the realism of MyDispense, which may be associated with student perceptions that MyDispense was more akin to a gaming platform. However, MyDispense was not built as a communication platform but rather to support the teaching of communication skills (for example in face-to-face learning activities), and perhaps this could be more clearly articulated to MyDispense users. Contrary to this, MyDispense was used as a replacement for experiential placements during the COVID-19 pandemic [7], with the intention to prepare students for future practice by exposing them to cases and scenarios that they would likely observe in real life. One such example is the incorporation of MyDispense within compounding laboratory classes which offered students an engaging simulated practice environment, which improved integration of various dispensing processes such as prescription dispensing and medication compounding, as opposed to considering them as segregated tasks [30]. Another example is the incorporation of a simulated safe for the storage and retrieval of controlled drugs and a drug register. Notably, the classification of such medicines differ from region to region, which for some MyDispense users limited its relevance and usability. It should be noted that MyDispense does not substitute theory or face-to-face interactions during placements and internships, which are imperative to student learning as they act as a stepping stone for novice practitioners to start their professional practice experience. Skipping this learning stage would mean that graduates will be ill experienced to handle the dynamic challenges of Pharmacy [31].

MyDispense is seen as a valuable asset to support pharmacy education, and most users interviewed had the intention to keep using the software, and to make use of its assessment capabilities. Some also mentioned their plans to collaborate with others to share cases and conduct research. We endeavor this will enhance engagement with MyDispense globally and most importantly improve the preparation of future pharmacists; our most important stakeholders. Of course, technology is not without its flaws, such as a lack of direct training available to users, particularly relating to building cases within the software, and this will need to be considered during future MyDispense software updates. Of the six MyDispense non-user institutions interviewed, five requested access to the software. Thus, the findings from this project will ultimately lead to strategies to put in place to overcome the various barriers that they (and other new users) may potentially face.

### Limitations

A limitation to this study is that there was only a small number of MyDispense non-users who agreed to participate. This may have limited the data available regarding other simulation software currently being used within pharmacy education globally. It is possible that non-users who were contacted about the study lacked interest in using MyDispense and may have been under the impression that they would be persuaded to purchase the unknowingly free software. Furthermore, although the results cannot be generalized to all MyDispense users, this study highlights factors that could be considered to improve usability and functionality and it also underlines attributes that new users could consider when implementing the software.

## Conclusion

The role of MyDispense and other dispensing simulation software and how these simulations were used in pharmacy programs was ascertained. Barriers and facilitators to MyDispense utilization, as well as future use and suggested improvements were identified, which will assist in the development of future enhancements, as well as instructions to assist smooth integration of MyDispense within pharmacy institutions.

## Supplementary Information

**Additional file 1. Appendix 1:** Themes, Subthemes and representative quotes identified from interviews with MyDispense users and non-users

**Additional file 2. Appendix 2:** Interview Questions for MyDispense users

**Additional file 3. Appendix 3:** Interview Questions for MyDispense Non-users

## Data Availability

The datasets used and/or analyzed during the current study are available from the corresponding author on reasonable request.
