# Supplementary material for: Barriers and facilitators to implementing simulation into pharmacy programs globally
Source: J Pharm Policy Pract. 2023 Feb 21;16:26. doi: 10.1186/s40545-023-00531-6 (PMC9943027; doi:10.1186/s40545-023-00531-6)
Supplement: Supplementary file 1 — Additional file 1. Appendix 1: Themes, Subthemes and representative quotes identified from interviews with MyDispense users and non-users [file 40545_2023_531_MOESM1_ESM.docx]

**Appendix 1: Themes, Subthemes and representative quotes identified from interviews with MyDispense users and non-users**

| **Main Theme** | **Subcategory (n Participants)** | **Exemplar Quote** |
| --- | --- | --- |
| Dispensing and counseling | Dispensing techniques (18) | “… I guess there's the more mundane side of the dispensing where it's product selection and label creation checking cal's, doing the scan, which is now requirement, or at least legally pharmacies are required to have this scanning technology and MyDispense has that built in” [01]  “What the patient should be having medicine wise and then they get their prescriptions and dispense them using MyDispense well they've got access to MyDispense so anytime we've released that, so in that way, they could have done it without doing the background” [02]  “We use the simulation of dispensing MyDispense to orient students to read prescriptions, taking in country consideration legality, safety, and other you know other evaluation skills in that first day. Suitability was the other word I was looking for it. Suitability for the patient is the other aspect we bring in” [04]  “For dispensing, for recognizing the errors at the prescriptions, how to deal with the patients, how to take the information from them. If you are able to recognize the drug- drug interactions in the prescription and so on.”[06]  “yep so right from day one in their first unit they start covering the law …, what needs to be on a label, what needs to be on a prescription so that starts in first year and is reinforced all the way through….”[14] |
|  | Time allocated to practice (13) | “We have regular tutorials and we do a series of six tutorials which integrate dispensing skills and then in the last tutorial we integrate communication skills around dispensing prescriptions as well, so information gathering as an example.” [04]  “Like the second year, when I suppose for a second year they're so new and we assume that they don't have jobs and pharmacy yet maybe some of them do, but a lot of them wouldn't have at that point, and so, and they have 3 three hour dispensing labs and three two primary healthcare workshops and so.” [09]  “They have like an hour in the seminar, and of course plenty of time at home to train and, as I heard some of the students also use it .” [12]  “But when it had its final collapse because all our systems are very old. And we didn't renew them because we had our teach out. So when it had its final collapse, last year we didn't restore that function. Because we sort of figured that got so much time in the lab. That they could practice as much as they wanted to yeah.” [14] |
|  | Other dispensing software (11) | “At this time in the degree we only use MyDispense because we can run in class simulations later in our degree, they progress to using purpose built to spend software in our dispensing.” [04]  “Students can always practice dispensing from home if they wish to, apart from the on-campus sessions, with the simulator provided by the government called FRED dispense. They can also do so through other software like EHR-go. You can dispense on EHR- go if you want to...” [07]  “And so there's two pieces of software that's commonly used in Community pharmacy And I don't know why we picked it over the other provider. We've been using…. the software that was used when I was an undergrad so it's more than 20 years, that we've been using that software so I’m not sure why we're still using it, or who decided that we’re going to use it, that just the one we use and its just rolled over.” [10]  “Well I inherited it… I guess, when the system was bought Fred was predominantly the one in the market. But you know, certainly for the new degree, if I was going to buy software again. I would look at something else yeah what else is in the market that's being used yeah.” [14]  ”No, they can't. One of the things that has been done in the past there's been options, where the dispensing laboratory has been accessible to students, just to do practice dispensing. We didn't do it this year because of time constraints and supervisor constraints, but yeah they can't access it remotely, they can only access it from that laboratory classroom.” [15] |
| Description of MyDispense use | Initial setup (8) | “It didn't come from the faculty, it didn't come from the school, I think. I think we had been looking for online options and I by word of mouth speaking with someone who coordinates in year four.”[01]  “we got all the thing sorted like with the IT department and sorting out the server yeah incorporated a server password and all that yeah” [03]  “I attended the workshop, so I put the information on the chat box below. It was held in Monash University Malaysia back in 2019 so that was my first exposure to MyDispense and so one day of intensive training.” [03]  “No. it's all approved within the school, but we are part of a greater Faculty of Medicine and health, so when it came to switching over to the new server I had to go up to a faculty level to get the support that I needed to do that kind of thing”. [04]  “I actually stumbled across you when I was doing research for writing this program and it looked really cool so I reached out…”[08] |
|  | Previously taught dispensing (6) | “So we have a dispensing system, we use Fred in person. Then we have dispensing labs, where they have a PC they have the scanner, a labeling printer and then the shelving with the medications on them so that's in person.” [01]  “So, before this what we did was of course come up with cases and, as I mentioned earlier on, we do have like a small dispensary room to try to simulate on the actual dispensary that we have in hospital settings and then we will proceed to do like on OSCE style so yeah that's how we've been doing our dispensary lessons yeah.” [03]  “So we never had a kind of simulation software before and before we had dispensing software like a pharmacy might use in actual practice…and so it was useful for kind of the process of making labels and but then everything else was extremely resource intensive so buying all the medicines there’s obviously a cost associated there and then they have a limited experience so we were finding more and more of our medicines were becoming expired.” [11]  “...before the pandemic we actually had an exercise, where the students did it live like they had to act. Some of them were pharmacists and others were patients and they acted it out, but, of course, you know during the pandemic that wasn't possible at all and we needed to find another exercise, and then we stumbled upon MyDispense but the students like it so much that we never changed it back.” [12] |
|  | Application (12) | “So the cases we are going to be built around hypertension, arrhythmias coronary heart disease and smoking cessation” [01]  “We cover dispensing, also prescription checking, calculations, responding to symptoms, yeah herbal medicines, even halal medicines are covered. We have some interesting use cases on our halal medicines and also on the medicine management during Ramadan yeah.” [03]  “In relation to MyDispense I use MyDispense in my dispensing subjects as well with hospital pharmacy in some situations… I do have plans on using it as well for pharmaceutical calculations, also in terms of prescription analysis as well. So those are my plans for MyDispense for pharmaceuticals.” [17] |
|  | Frequency of use (6) | “MyDispense is started from day one of each rotation until the end of the rotation.” [06]  “In the timetable there's a specific allocated time allocated for this module so normally on a Tuesday morning. Umm yeah, it's so it's so the module runs every Tuesday morning but we do start off with lectures first sort of equip them before we actually go on to do the workshop cases, yes.” [03] |
|  | Dispensing assessments (10) | “... with MyDispense there's like that simulated patient that incorporates that communication skills. So, before this our OSCE was that communication skills separate to dispensing skill set…so I think it's beneficial to have this it's more added value to have this MyDispense software implemented in our teaching and learning module because of the additional skills that the student can be taught and practice, actually, even if you dispense it wrong is still don't kill the patient right.” [03]  “So when we originally started doing examinations online with students conducting examinations from a remote location, we couldn't authorize access to the MyDispense server. Because it was external to the system, the proctoring system, a website and external it's a separate server to the University Canvas, which is where most of our quizzes in our learning management system where most of the assessments are situated. So we then had to make a workaround for how to get the material from. To be able to transfer that prescription material in a way that was going to provide a meaningful assessment, because I couldn't allow the students to use MyDispense during the exam.” [04]  “ They have a calculation assessment… some of that is prescription based, and they have two OSCEs and part of the OSCE assessment is well, to OSCE itself, so the communication aspect but there's also an aspect where they need to clinically validate prescriptions as well and most of the assessments that I’ve been involved with it has mostly been around clinical validation of the prescription …previously they have had to go through the full dispensing processes when they build a prescription and generate a label and dispense the product” [10] |
| Barriers to MyDispense use | University costs (8) | “And she hasn't had a huge amount of time. Also she's pretty understaffed but she's been using master’s students to actually assist with the development of things and scenarios in it.” [05]  “Well we use a subject matter expert to help create the exercises so there's definitely a cost in that, I wouldn't know how to break that out because we paid her a lump sum to develop our whole program. So I mean I guess you could calculate her time in my time with regards to setting it up, but as far as I know, that was our only fee.” [08]  “When we started off, I think our Internet was a bit overwhelmed as for the server I guess was overwhelmed with the number of students that were taking are using MyDispense at the same time…So, in terms of upgrades I think we did have to shell out some of technicalities in terms of increasing bandwidth upgrading internet and facilities to cover all of the students using the internet, at the same time” [17] |
|  | Academic staff time (6) | “For us, No, I mean there's I guess if you put down staff time, yeah this is a human resource.” [01]  “Every time we update it, I think it takes it probably about eight hours for new drug to be done and it probably for each new drug and if we're doing five drugs, and then we have to update all the ones that we keep the same they will take less time…” [02]  “Not from a monetary point but I suppose one of the things that really needs a champion and we have had a champion in our department of actually doing the graft around us and, so you need people, and the people need time and this is probably the most stretched resource really.” [09] |
|  | Lack of training (4) | “I think it’s a bit tricky with the signatures, the doctor signatures like trying to actually get it to the right, the right size, with a good resolution. That was a bit fiddly but yeah managed to work it out, in the end.” [01]  “… because it's a digital program you know when we start, we have this great let's do this and then you realize that you've made an error go back and fix it up, so that all takes a lot of time and that's extensive.” [02]  “..I think the second thing would be expanding the portfolio of medications that we can make script prescriptions from…for example, sometimes we're looking for a generic alternative to demonstrate that difference in from the known brand to perhaps a generic and there’s not always a generic in the system and then to actually set that up…we've had to seek help because of the images that are required and the scanning and those sorts of things for the higher levels of the course which is more sophisticated…I have found that cumbersome if it's not already in the system.” [04] |
|  | General difficulty in use (8) | “So when we originally started doing examinations online with students conducting examinations from a remote location, we couldn't authorize access to the MyDispense server. Because it was external to the proctoring system, a website and external it's a separate server to the University … So we then had to make a workaround for how to get the material to be able to transfer that prescription material in a way that was going to provide a meaningful assessment, because I couldn't allow the students to use MyDispense during the exam.” [04]  “I do find the enrollment of students a little bit cumbersome. So, there's a multi-layered system within the system where you need to enroll them in MyDispense and then enroll them in units.” [04]  “… we will see a lot of our students have very poor computer literacy. But they do computer literacy course in first year” [05]  “My students tend to struggle going through the simulations and the complexity of it, I believe, so we have to do a lot of walkthroughs with student’s kind of showing them, you know, speaking with them on the phone.” [08]  “So that we did have in 2020 some feedback that they thought it was a game. We gave them videos and PDFs of how to access and how to navigate MyDispense, but because they saw the avatars and things they thought it was a game, they didn’t really take it seriously and they didn't understand how to optimize it and they didn't even get to the feedback PDF at the end.” [09] |
|  | Lack of collaboration (7) | “I understood that there was an ability to share cases. I've never really gone down that track so I’ve not actually done the mechanics of how that will work but yeah.” [02]  “I suppose it would be a resource thing, I would have to talk to the powers above to see if they will be willing for us to share. Because certainly building up that product bank and all the images like I guess it's been a barrier to using it more, and so, if there was some way that you could split that sort of stuff with another provider there would be some benefits to that....” [09]] |
|  | Lack of realism (3) | “MyDispense has definitely helped. I think there are benefits to keeping it as a supplementary activity. I think for our assessment, I think we need to move back to simulating. You know the actual practice of dispensing skills and counseling and face to face as soon as possible.” [01]  “Pharmacy Sam is nearer to what we are doing, because they are like a collaboration and interaction among the health professionals.” [06]  “Yeah, I think the skills are similar, I think the only thing that's different is the information gathering or communication with the patient or the prescriber it's probably not…like artificial intelligence, where you're talking to a virtual patient and they will respond based on your question, you can just see the questions… that's sort of …a bit limiting.” [09] |
|  | Difference in jurisdiction and drug nomenclature (4) | “Think there's been some harmonization with drug names, so I noticed that I started going in and I was thinking about changing them….Anyway, there's a few of them in there that are still with a different spelling. But we're trying to update all of our content, so that it's in agreement with the global position on drug names.” [01]  “I think, there's a few things about the poisons regulations which are different in Queensland too…there's a disagreement between them to not do it so it's just kind of working around that going well that's just a small work around anyway, they might work in another state, so they need to get used to these ideas and our laws are changing.” [02]  “When I took over everything was Melbourne addresses or Victorian doctors. Which from a jurisdiction and legal point of view, has some implications, especially when we start talking about controlled substances…” [04] |
|  | Sustainability (4) | “… with some colleagues that they have actually resisted using it, even though they're very aware of it, and that they get good reports from year four… being concerned that you know you don't invest too much time because you know you don't know what Monash might do, it's not ours. You know, maybe they bring in a fee or subscription or some of some kind.” [01]  “And yeah, I mean everybody at the university when we've spoken about it and I’ve been teaching and learning and forums and they you know they will say, and this is free, and you know they're all very … concerned about the sustainability, how long this is going to remain free.” [05]  “…sometimes you know when you sign up for things in there, like six months free for free and then you like, are you going to pay the updates and then you put a paid for this and you could pay for that, so I was just checking” [10] |
| Facilitators to use MyDispense | Cost (7) | “I loved it, so I was like if they’re offering this for free I’m going to take it, and you know it has Monash all over it so there's some free advertising in there but the students don't mind, I think. it's just an excellent product and I can't really believe it's kept free and that you're able to do that, that goodwill it's just excellent.” [01]  “To be honest, it’s interesting, it's free. I don't have let you know when you want to subscribe to something that commitment to pay them normally. We are national university is also hard for us to buy something new…” [03]  “So we don't use the old dispensing software anymore, and it was quite expensive as well. That was another plus side of MyDispense obviously being free software is that it freed up resources for us to use elsewhere …” [11] |
|  | Training (9) | “Okay, I see we are kind of taking not shortcut but because to develop their own case it's very time consuming so we wrote to a MyDispense user…as he is also a trainer for my dispense so he kind of helped us to set up the framework so that we can start importing our local settings our Doctor’s, our own regulations, so I the whole process in that way was expedited, so it takes two months...” [03]  “We found the support that we've had from the MyDispense team absolutely amazing. You know that they bend over backwards to assist us. What we have since done as well is really to share it with other colleagues…” [05]  “ So every batch… one day for them to teach them like, as you said, a lecture on how to deal with MyDispense. Starting from how to enter into the database and how to give them the password, the username and so on. And we practice and train them, step by step, this is the computer screen, how to search the patient's name, and history, and this is how to check for the prescription, this is how to check for drug interactions. This is how to go through the questions for the patient and the questions for the doctors as well…” [06] |
|  | Promotes collaboration (8) | “ I realized, it is quite collaborative, yeah.” [01]  “I must have gone to a MyDispense thing at Monash and I feel like that was attached to the APSA Conference so that was, I think, 2019. So that was where I learned about that…” [02]  “We could extend that collaboration to research. This could open up many opportunities for us as well, so from just one software it could. It could link us to other countries and also other opportunities, I think so that's a way forward” [03] |
|  | Realistic environment (10) | “It's an in-classroom tool that can actually orient them before they go out on placement, before they can get exposure to the labs. On some of those really fundamental principles that underpin the profession and professional standards, so we can actually do that in life, like exercises, which I think is a lot more powerful.” [04]  “I think it gives them a feel for the real-world pharmacy a little bit in terms of you know kind of what to expect situations that might arise as they work in a pharmacy. You know, as with any simulation not all aspects are going to be able to be simulated but, for the most part, I think it does a pretty good job...” [08]  “We kind of use the simulation across and placements, depending on the situation, whether we can get students out placement, so we have used MyDispense quite a lot obviously over the last three years…we recreated the Community pharmacy setting in gave them dispensing exercises and checking dispensing, so validation exercises, primary health care exercises… so yeah we used the different methods with different types of exercises, with MyDispense.” [09]  “As a preparation just really just before they start their internship So they don't only use it in the seminar but also after that.” [12] |
|  | Controlled drug function (2) | “… my favorited part is the drug part, so I like how they will separate them, you know controls and non-controlled. Because that's very much so, how it is in the pharmacy and so there is like a safe, where they keep you know your controlled substances and the students will have to know ‘Oh, this is a controlled substance’, I need to get it from the safe.” [08]  “We did a control drug and I really loved the way they're able to go and click on the safe and open the safe, it just kind of got them on the idea that it's not just selecting something off the shelf, and they filled out the control drugs register and although it looks different from New Zealand, it was the process of knowing that there's documentation that goes with that and that's why I really wanted to use it and particularly this oncology lab that I was doing where they were dispensing morphine, just to give them that experience and have been thinking about those steps that are required in that situation.” [09] |
|  | COVID (9) | “So it was probably partly COVID I don't think there was really any other push for being online... COVID created the opportunity and then Monash had the asset so yeah.” [01]  “At least we have found a solution during the pandemic area…at least, we found the solution, other universities are still struggling at that point.” [06]  “I suppose we’ve really utilized it in three ways. Giving us that extra opportunity to practice as I outlined in a workshop we ran this week, we've utilized it within a classroom face to face setting and then, when we've not been able to have placements run as a normal program… due to the impact of the pandemic we've used it as a replacement as a supplement and replacement really face to face learning, so I suppose we’ve kind of dipped in and out and used it in different ways.” [09] |
|  | Cultural representation (5) | “ We introduced the halal medicine cases only this semester. We wanted to expose different cultural experiences to the Philippines students, so I think the students felt like it was very relevant... It’s nice that they incorporated people with scarf I mean on my perspective, so it's good like oh, I want to talk about a Muslim, so I want an appropriate picture to incorporate so when trying to find the right icon or Avatar …you provide it is more inclusive I find so that's kind of nice and like being I’m a Muslim myself, so if I want to add some cultural counselling in a way for our setting so that's what I like about MyDispense well.” [03]  “We’ve got the Welsh prescriptions on there now, which is new in the last couple of years… but there's not really been any downside to using MyDispense so that's great” [11] |
|  | Easy to use (7) | “I think I looked at your online video as well, there were some helpful online videos I think I went to and I watched a few of those to you know pick up my skills, but no, no formal training, it's quite user friendly.” [01]  “Yeah, fair to say because it is also quite user friendly, we were able to overcome the whole initiative cuts that we are facing. And also, good to know that the internet consumption or the bandwidth required for MyDispense is not too heavy. So students still have no have no issues accessing.” [03] |
|  | Engaging experience (3) | “I guess anecdotally I think they’ve liked it… I think, to me, the actual engagement is the key like you know I’m actually seeing the students both externally and internally logging in and tapping through those activities, and I think that's probably the key driver that I’m getting you know a good level of participation.” [01]  “We go through the same thing they role play. We get peer feedback, we get staff feedback, but the depth of interaction in the seminar is much improved. The students have to have completed the MyDispense scenario. They have to have uploaded a PDF demonstrating they've completed it, whereas previously they prepare the work they come in. Some were prepared, some weren’t prepared. “[05] |
|  | Saves staff time (6) | “... once we've got the scenarios set up and running, there are things that you can do that free us to do other things in terms of...On one level it obviously takes staff input in the beginning, but once it's up and running, it frees staff actually to do other things yeah.” [05]  “... the feedback part of it is really time consuming for us as teaching staff. So for them to get the answer, rather than having me having to tell them the answer and it just takes out of it at time factor is you know there's a lot of skills that they were getting from it, that we just couldn't we couldn't really replicate that. [09]  “… Just imagine if you teach pharmaceutical calculations and you need to go over to each student under calculations right, this is the same thing and dispensing and you need to you need to read, each one and give feedback for each student okay, and this could be done in MyDispense you could personally give your insights your feedback to your students, based on their answers and you could also communicate to them on hints or reinforcements on the given case so that's really a good thing it really helps a lot and that's what really I like with MyDispense.” [17] |
|  | Student feedback (8) | “… great feedback, positive feedback. They really enjoy using it …They really enjoyed the fact that it felt quite like a life kind of scenario … we introduced on the halal medicine cases only this semester, we wanted to expose different cultural experiences on to the Philippines students, so I think the student felt like it was very relevant, very they can learn a lot from these activities, so we share nothing but positive feedback on from the session actually…[03]  “The student feedback certainly seems like they enjoy it.” [04] |
| Future use of MyDispense | Plans (9) | “You know that there are little things that we are busy speaking with the MyDispense team about. You know, we have OTC's from behind the counter. And I know they have the under the counter and they have the front shop, so there are those kinds of things that we're talking about. We do a lot of point of care testing.” [05]  “The next thing that we, we can do is that maybe we can introduce this also to the pharmacy practitioners, because I think this can really influence them later on in terms of our pharmacy practice in the Philippines, so it's like having all those patients recording and also having all those that we didn't actually practice here in the Philippines, so we can actually have up there, later on.” [17] |
|  | Suggested improvements (9) | “guess it would be good to have been able to put in the rule to match down control drugs, because you know that would mean that you've got a default rule and that you can’t change that too much.” [02]  “I feel like we’re not updated often enough, so I think I feel like the product is developing, perhaps the depth of the activities are developing and I don't know about it, so I think that if I had to change anything and it's not about software it's about more about the two way communication between the MyDispense team and the institutions using it. is, I think we need to have a little bit more of an understanding of the changes that have been made in scope and that sort of thing. That would be one thing I would improve. Yeah it doesn't even have to be in person and a newsletter even bulletin ones per semester.” [04]  “Do you have like a dispensary full of generic products… like you know no frills packaging type of you know here's your morphine, your carbamazepine and every institution or every country could pick and choose what they have available, that could be something they could do.” [09] |
