# Supplementary material for: Barriers and facilitators to implementing simulation into pharmacy programs globally
Source: J Pharm Policy Pract. 2023 Feb 21;16:26. doi: 10.1186/s40545-023-00531-6 (PMC9943027; doi:10.1186/s40545-023-00531-6)
Supplement: Supplementary file 2 — Additional file 2. Appendix 2: Interview Questions for MyDispense users [file 40545_2023_531_MOESM2_ESM.docx]

**Interview Questions for MyDispense users (adapted from Phanudulkitti et al. (2021))**

**We would like to know how you teach dispensing skills using MyDispense to your students.**

1. What is the name of the course/unit in which students are taught dispensing or practise dispensing?
2. How many students are enrolled in this course/unit?
3. What year do students undertake this course/unit?
4. How do you teach dispensing skills OR how do students practise dispensing? *(the question asked will depend on response to Q1)*
   1. Can you describe the methods that you use?
5. What opportunities do you give students to practice their skills?
   1. How much time is allocated to the modalities outlined above?
   2. How often do you use dispensing software?
6. How do you use MyDispense as part of these sessions?
7. What made you decide to use MyDispense? How long has it been implemented?
8. Could you describe the initial set-up of MyDispense?
   1. MyDispense as software is free, but were there any unforeseen or additional monetary costs that you can think of?
   2. Are there any ongoing costs?
   3. What internal or external resources were required to set this up?
   4. Were there any governance bodies that you had to consult?
9. How long did it take, in terms of time to get the software up and running for students?
10. What topics do students learn using MyDispense?
11. What skills do students learn using MyDispense?
12. Do you use MyDispense to assess skills and competencies? If so, how?
13. Did you/ the teaching staff need to be trained to use MyDispense?
    1. What did the training involve?
    2. How long was the training?
    3. Who provided the initial training?
    4. Who provides the ongoing training?
14. Do you share the simulation activities/ assessments with academic staff at other institutes?
    1. why/ why not?
15. Have you faced any challenges using MyDispense?
    1. If yes, what were they?
    2. How have these been overcome?
    3. Which challenges have not been overcome?
16. What do you like about MyDispense?
17. Is there anything that you would change about the MyDispense to make it better suited to your needs?
18. Is there anything else you would like to mention regarding the simulation software?
