# Supplementary material for: Barriers and facilitators to implementing simulation into pharmacy programs globally
Source: J Pharm Policy Pract. 2023 Feb 21;16:26. doi: 10.1186/s40545-023-00531-6 (PMC9943027; doi:10.1186/s40545-023-00531-6)
Supplement: Supplementary file 3 — Additional file 3. Appendix 3: Interview Questions for MyDispense Non-users [file 40545_2023_531_MOESM3_ESM.docx]

**Interview Questions MyDispense Non-users (adapted from Phanudulkitti et al. (2021))**

**We would like to know how you teach dispensing skills to your students.**

1. What is the name of the course/unit in which students are taught dispensing or practise dispensing?
2. How many students are enrolled in this course/unit?
3. What year do students undertake this course/unit?
4. How do you teach dispensing skills OR how do students practice dispensing? *(the question asked will depend on response to Q1)*
   1. Can you describe the methods that you use?
5. What opportunities do you give students to practice their skills?
   1. How much time is allocated to the modalities outlined above?
6. How often do you use dispensing practice exercises in your unit?
   1. Approximately, how many hours would that be?
7. Do you use simulation as part of these sessions?

*If yes,*

1. What program/software do you use?
2. What made you decide to use this program/software? How long has it been implemented?
3. Could you describe the initial set-up of the program/software?
   1. What internal or external resources were required to set this up?
   2. Were there any governance bodies that you had to consult?
4. Did you pay for the program/software?
   1. Are there any ongoing costs?
5. How long did it take, in terms of time to get the software up and running for students?
6. How is it accessed by students?
7. Can students access the program remotely?
8. What topics do students learn using the program/software?
9. What skills do students learn using the program/software?
10. How to you assess those skills and competencies (outlined above)
    1. Do you use the software to do so? If so, how?
11. Did you/ the teaching staff need to be trained to use the program/software?
    1. What did the training involve?
    2. How long was the training?
    3. Who provided the initial training?
    4. Who provides the ongoing training?
12. Do you share the simulation activities/ assessments with academic staff at other institutes?
    1. why/ why not?
13. Have you faced any challenges using the program/software?
    1. If yes, what were they?
    2. How have these been overcome?
    3. Which challenges have not been overcome?
14. What do you like about the simulation software/ program?
15. Is there anything that you would change about the program/software to make it better suited to your needs?
16. Is there anything else you would like to mention regarding the simulation software?

MyDispense:

11. You mentioned some of the problems/ challenges you faced with your current dispensing software…. Did you know that Monash University offers a free web application called MyDispense?

Some of the things the program offers include:

- Dispensing skills and product selection
- OTC counselling
- Prioritisation of tasks such as dispensing, taking phone calls, and interacting with patients
- DD book interactions
- Immediate contextual feedback is provided to students after completing tasks
- Teaching staff from other intuitions can share tried and tested assessment cases- so you don’t have to spend time making your own
- It's also free

If yes,

1. Why did you decide not to implement the software at your institution?

If no,

1. Would you consider implementing the software now that you know more about it?
2. Do you have any questions?
3. Is there anything else that you would like to mention regarding dispensing the use of simulation or MyDispense?
